# Super-condenser enables labelfree nanoscopy

**DOI:** 10.1364/OE.27.025280

**Published:** 2019-08-22

**Authors:** Florian Ströhl, Ida S. Opstad, Jean-Claude Tinguely, Firehun T. Dullo, Ioanna Mela, Johannes W. M. Osterrieth, Balpreet S. Ahluwalia, Clemens F. Kaminski

**Affiliations:** 1 Department of Physics and Technology, UiT The Arctic University of Norway, NO-9037 Tromsø, Norway; 2 Department of Chemical Engineering and Biotechnology, University of Cambridge, CB3 0AS Cambridge, United Kingdom

## Abstract

Labelfree nanoscopy encompasses optical imaging with a resolution in the 100-nm range using visible wavelengths. Here, we present a labelfree nanoscopy method that combines coherent imaging techniques with waveguide microscopy to realize a *super-condenser* featuring maximally inclined coherent darkfield illumination with artificially stretched wave vectors due to large refractive indices of the employed Si_3_N_4_ waveguide material. We produce the required coherent plane wave illumination for Fourier ptychography over imaging areas 400 μm^2^ in size via adiabatically tapered single-mode waveguides and tackle the overlap constraints of the Fourier ptychography phase retrieval algorithm two-fold: first, the directionality of the illumination wave vector is changed sequentially via a multiplexed input structure of the waveguide chip layout, and second, the wave vector modulus is shortend via step-wise increases of the illumination light wavelength over the visible spectrum. We test the method in simulations and in experiments and provide details on the underlying image formation theory as well as the reconstruction algorithm. While the generated Fourier ptychography reconstructions are found to be prone to image artefacts, an alternative coherent imaging method, rotating coherent scattering microscopy (ROCS), is found to be more robust against artefacts but with less achievable resolution.

## 1. Introduction

Conventional nanoscopy, optical microscopy with resolution below 100 nm, is based on fluorescence [1]. Often listed advantages of nanoscopy, especially in comparison to electron microscopy, are the simple sample preparation, live-cell compatibility, and molecular specificity. Though live-cell compatible, the introduction of fluorescent labels onto the molecular structures of interest are in living cells likely to cause both functional and structural aberrations, potentially leading to false conclusions, and is also associated with problems like photobleaching and phototoxicity, variable label specificity, imaging- and image reconstruction-related artifacts, and lengthy optimization protocols [2, 3]. The advantage of label specificity also has its downside of excluding (ultra)-structural context of the specifically labeled structure, although this can be alleviated to some degree via multi-channel labeling. Synergistic approaches combining the advantages of label specificity from conventional nanoscopy together with ultra-structural context obtained via labelfree nanoscopy, could bring many new insights about cellular functions, especially as (contrary to correlative light and electron microscopy [4]), labelfree (optical) nanoscopy has the potential of also being applied to living cells and cellular systems. Excluding methods that have not gone beyond the proof-of-principle stage like hyperlensing [5] or super-oscillation microscopy [6], suitable labelfree methods that have the potential to provide nanoscopic resolution can be sorted broadly into four groups:

1. *Autofluorescence probed with conventional nanoscopy*. Although certain critical fluorophore properties that are required for ultra-high resolution nanoscopy like photo-switching [7] are normally not present in intrinsically fluorescent samples, structured illumination microscopy has been shown to resolve features in the 150 nm regime in unlabeled retinal tissue [8]. Note that intrinsic fluorescence is a property present only in some but not all samples.
2. *Nearfield scanning optical microscopy* [9], which rasters a sample with an effective resolution below 100 nm. Akin to electron microscopy, this scanning optical approach has a low through-put and is challenging to combine with fluorescence-based nanoscopy.
3. *Deep ultra-violet microscopy* - a theoretically simple approach as resolution scales linearly with employed imaging wavelength. However, the limited availability and performance of optical components in this spectral range as well as the high phototoxicity associated with ultraviolet radiation offset the benefits gained by wavelengths below 400 nm illumination.
4. *Fourier ptychography*, FP [10], a technique specifically developed for improving digital pathology [11].

In FP the sample is illuminated and imaged sequentially with plane waves from a multitude of directions that densely sample the illumination condenser numerical aperture (*NA_c_*). The generated set of images is then synthesized into a super-resolved amplitude and phase image of resolution Δ*x* given by

(1) $$\Delta x=\frac{\lambda }{{\mathrm{NA}}_{c}+{\mathrm{NA}}_{o}}$$

via a dedicated *phase retrieval algorithm* [10]. It is well known that in conventional microscopy the condenser *NA_c_* used for illumination should be matched to (or even slightly below) the objective *NA_o_* [12], resulting in an effective maximal resolution of $\Delta x=\frac{\lambda }{2{\mathrm{NA}}_{o}}$, the Abbe resolution limit [12]. Crucially in FP, a numerical aperture *NA_c_* of the condenser *can* be larger than the objective’s *NA_o_* in order to increase resolution with respect to the detection objective.

## 2. Theory

**Fig. 1:**
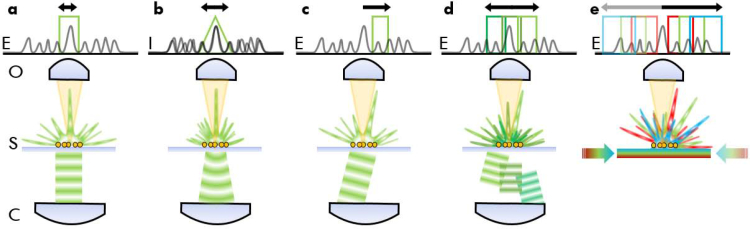
Amplitude/modulation transfer functions using (a) coherent, (b) incoherent, and (c) oblique illumination. Amplitude transfer function sampling in (d) conventional FP and (e) waveguide-based Fourier ptychographic microscopy. E: electric field, I: intensity, O: objective, S: sample, and C: condenser. The arrow highlights the cut-off frequency for different imaging modalities.

As the illumination in FP is coherent, the complex field (amplitude and phase) of the sample is probed rather than its intensity as in incoherent imaging. Even though the effective aperture of a coherent microscope is half the size of an incoherent one’s, plane wave illumination at oblique angles re-positions the sample’s field in the aperture, thus giving access to finer details as visualized in Fig. 1a–c

. Further, because the down-modulation of sample spatial frequency information with the illumination’s spatial frequency occurs before being low-pass filtered by the objective aperture, access to information beyond 2*NA_o_* is possible given high lateral spatial frequency of the illumination at greater angles than conventionally associated with *NA_o_*. To extract those finer details, multiple images are acquired sequentially using illumination angles spanning the entire condenser *NA_c_*, and combined into a super-resolved image computationally (see Fig. 1d). Despite its potential, an extended condenser *NA_c_* has so far almost exclusively been used to increase the space-bandwidth product [10] rather than performing nanoscopy. This is because, assuming the highest *NA* available for both illumination and detection, the resolution caps at the incoherent resolution limit and is hence in the orderhttps://www.overleaf.com/project/ of 200 nm. The largest illumination *NA* so far was demonstrated using an oil immersion condenser featuring an *NA* of 1.2 [13]. To resolve nanoscopic structures using FP, a *super-condenser* allowing illumination with spatial frequencies exceeding those offered by the best immersion objectives is necessary. In the following, we show how such a super-condenser can be implemented in the form of a photonic waveguide chip in conjunction with multi-spectral illumination. We show simulation results of the proposed method and perform proof-of-concept imaging of sub-diffraction-limit sized metal-organic framework (MOF) clusters. Figure 1e outlines the fundamental mechanism of the proposed super-condenser, which aims to optimize the magnitude of the lateral illumination wave vector components and to provide as dense coverage of the virtual condenser pupil as possible. To achieve largest lateral wave vector components, the illumination administered to the sample via photonic waveguides is intrinsically orthogonal to the detection objective and thus allows to maximize the wave vector magnitude geometrically. Furthermore, akin to microscopy with immersion media, the illumination wave vector is stretched by a factor determined by the refractive index of the waveguide material. Thus, by apt choice of material, a further tremendous increase in wave vector magnitude and image resolution can be achieved. To illustrate, a conventional fluorescence microscope imaging GFP (*λ*_*ex*/*em*_ = 488/512 nm) with a high-performance 0.95 NA air objective offers a maximal resolution of *λ_em_*/2*NA_o_* ≈ 270 nm. The same sample imaged with the proposed super-condenser featuring Si_3_N_4_ waveguides with refractive index n ≈ 2.08 yields a theoretical resolution of *λ_ex_*/(n+*NA_o_*) ≈ 160 nm. Furthermore, it should be considered that incoherent microscopy techniques (like fluorescence or brightfield) have a strongly damped optical transfer efficiency for higher spatial frequencies, whereas the effective transfer function produced by Fourier ptychography has close to unity transmission strength, thus obtaining greatly enhanced contrast for finer structural details (as visualised in Fig. 1) [14]. This feature is mainly used by an alternative coherent imaging technique, rotating coherent scattering microscopy (ROCS) [14], which averages over multiple azimuthal orientations to mainly increase contrast. The benefit of ROCS is then its robustness against reconstruction artefacts, which render the technique an interesting alternative to FP as is shown below.

## 3. Methods

### 3.1. Waveguide design

**Fig. 2:**
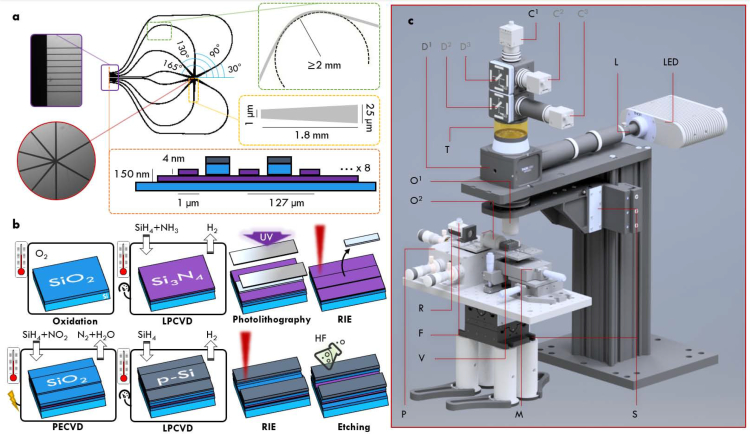
(a) Chip design: 8 inputs deliver visible light at various illumination angles to the imaging region, while simultaneously ensuring single-mode characteristics through bending (bend radii ≥ 2 mm) and adiabatic tapering. (b) Waveguide production steps: the surface of a silicon waver is thermally oxidized and subsequently covered with a layer of silicon nitride via low-pressure chemical vapor deposition (LPCVD). The waveguides structure is then created via photolithography and reactive ion etching (RIE) to produce the required 4 nm-sized rib. A protective wall between the waveguides is created via plasma-enhanced CVD of silicon oxide followed by LPCVD of polycrystalline silicon. RIE followed by chemical etching using hydrofluoric acid (HF) uncovers the waveguides again [15]. (c) The optical microscope as outlined in the main text: LED illuminator (LED), liquid light guide (L), fibre input for lasers (F), reflective collimator (R), vacuum stage (V), piezo stage (P), micrometer stage (M), sample stage (S), objectives (O^1/2^), tube lens (T), (dichroic) mirrors (D^1/2/3^), cameras (C^1/2/3^).

Photonic integrated systems out of high-index contrast materials have been used for various applications over the last decade and have recently been developed further for sensing tasks in the visible wavelength range [16–25]. Building on this previous work, the super-condenser waveguides were designed to provide a high refractive index (>2) and sufficiently high-intensity single-mode illumination (in the mW range) of multiple visible wavelengths over a large area (>100 μm^2^), which can be switched between several distinct directions spanning a full circle. A sketch providing an overview and details on the waveguide geometry are presented in Figure 2a

. Si_3_N_4_ was used as guiding material in rib waveguide geometry [18, 19]. A total slab thickness of 150 nm was chosen, which realizes a beneficial trade-off between reach of the evanescent field and coupling efficiency (coupling efficiency increases with waveguide thickness, whereas the evanescent field penetration depth decreases). To ensure a homogeneous field distribution over the waveguide surface while simultaneously keeping the guided light as mono-directional as possible, the waveguide geometry was designed to fulfil single-mode conditions [15, 26]. The necessary waveguide design was carried out using the software package FIMMWAVE (Photon Design, Oxford, UK). In addition to optimising the slab thickness, simulations at wavelengths spanning the visible region (488 nm, 561 nm, 660 nm) showed that a 4 nm etched rib is necessary to enable single-mode condition at 488 nm, with larger wavelengths being feasible at taller rib heights [15]. Hence, an etched rib height of 4 nm was chosen. In width, the waveguide guide structures were limited to be no smaller than 1 μm, allowing the use of conventional photolithography with homogeneous results over a full 4” wafer, while still providing single-mode characteristics after coupling. Adjacent to the coupling region on the chip edge, the waveguides are designed to broaden out to enable larger fields-of-view. To maintain the initial single-mode conditions, broadening to a waveguide width of 25 μm was performed via adiabatic tapering with a linear taper of 1.8 mm length, which was found to achieve a high guiding efficiency (>90%) for all used wavelengths [26]. To enable illumination from multiple directions, eight inputs with a spacing of 127 μm were realized that bend towards a common imaging area, with no bend radius smaller than 2 mm. Simulations showed that less than 1 dB loss at 90° for a 2 mm bend radius occurs for 488 nm and 561 nm, while 660 nm light is attenuated by ∼3.5 dB. The spacing of 127 μm was set between the arms to match conventional fiber-array adaptors that are standard in the telecommunication industry and could allow for fastest switching between the waveguide inputs in future set-ups. Since the guided light is not tightly confined to the rib due to its small dimensions, slab propagation is prone to occur at the chip input and at curvatures. The light guided in the slab can create cross-talk between neighboring rib structures, reducing the contrast between the illumination angles. This is avoided by a layer between the rib structures consisting of 200 nm of SiO_2_ with 100 nm of polycrystalline silicon (p-Si) on top. The high-refractive index of p-Si effectively diverts the light coupled at the slab to itself, preventing it from leaking into the neighbored ribs. This absorbing layer follows along the rib structures, with a constant gap of 5 μm to the rib wall.

### 3.2. Production steps

Waveguide chips were produced at the Institute of Microelectronics Barcelona (IMB-CNM, Spain). The essential production steps are summarized in Figure 2b and details of the fabrication optimization and process can be found elsewhere [27]. In short, a silica layer with a thickness of 2 μm was first grown thermally on a silicon chip, followed by the deposition of a Si_3_N_4_ layer using low-pressure chemical vapor deposition (LPCVD) at 800°C. Then, standard photolithography was employed to define the waveguide geometry using photoresist, followed by reactive ion etching (RIE) to fabricate the delicate 4 nm height of the waveguide rib necessary for single-mode guiding. This can be structured into four sub-steps: (1) resist spinning on the continuous Si_3_N_4_ layer; (2) light exposure through a mask and chemical development to remove resist on non-desired areas around the waveguide structures; (3) RIE to desired etch depth; and (4) removal of remaining resist through plasma ashing and solvent washing. After removing the remaining photoresist the absorbing wall between the rib structures was created. For this 200 nm of SiO_2_ were deposited by plasma-enhanced chemical vapour depostion (PECVD) followed by 100 nm of polycrystalline silicon deposited by LPCVD. RIE removed the the p-Si and ca. 190 nm of the SiO_2_ above the waveguide structures, with the remaining SiO_2_ being etched away using hydrofluoric acid to prevent damage to the rib structures.

### 3.3. Optical microscope

For experiments, a custom-built upright microscope was used, which was described in detail elsewhere [28]. A 3D model of the system is shown in Fig. 2c and basic features are be reviewed here briefly. The microscope was based on a modular commercial system (CERNA, Thorlabs) and offers up to four LEDs with wavelengths centred at 385 nm, 490 nm, 565 nm, and 625 nm for episcopic illumination of the sample. The illumination light was produced in a four channel LED combiner (LED4D245, Thorlabs) and delivered to the main frame via a liquid light guide of 3 mm diameter (LLG0338-4, Thorlabs). To counter the broad emission of the 565 nm LED, a bandpass filter (#86-986, Edmund Optics) was installed in the LED combiner. The light guide output was focused using two lenses (AL2018-A and LBF254-040-A, Thorlabs) onto the front focal plane of a collimator lens (LBF254-040-A, Thorlabs), where an iris (SM1D12D, Thorlabs) was used to create Köhler illumination. Using a tube lens (LBF254-100-A, Thorlabs) in a 4f-system with the collimator lens, the LED illumination was then focused via a semi-transparent mirror (which was removed when using evanescent chip-illumination) onto the back focal plane of the objective lens (UPLSAPO40X2, Olympus).

Evanescent illumination of the sample was achieved by focusing laser light into the waveguide chip inputs. Three laser lines at 488 nm with 150 mW (OBIS 488LS, Coherent), 561 nm with 150 mW (OBIS 561LS, Coherent), and 647 nm with 120 mW (OBIS 647LX, Coherent) were used and custom combined using dichroic mirrors (ZT514rdc and ZT594rdc, Chroma) and coupled into a single-mode fibre (S405-XP-custom, Thorlabs) using a commercial fiber coupler (PAF2-A4A, Thorlabs). The fiber output was collimated using a reflective collimator (RC04FC-P01, Thorlabs) and focused with a 50× 0.5NA objective (LMPLFLN-BD 50X, Olympus) onto the inputs of the waveguide chip. To aid coupling into the single-mode waveguides, the reflective collimator and the focusing objective were installed on a differential micrometer stage with additional piezo fine controls (MDT630B/M with MAX302/M, Thorlabs).

The chip itself was resting on a vacuum stage (HWV001) and held in place with low vacuum. A three-axis long travel block (RB13M/M, Thorlabs) allowed coarse alignment of the chip with respect to the differential micrometer stage and lateral translation of the whole assembly was realized via a motorized two-axis translation stage (PLS-XY, Thorlabs). Focusing onto the sample was achieved by translating the imaging objective via a 1 inch travel module (ZFM2020, Thorlabs) with micrometer precision. Finally, the light captured by the imaging objective was focused by a tube lens (TTL180-A, Thorlabs) onto a CMOS camera (UI-3080CP-M-GL Rev.2, IDS), which featured a 3.45 μm pixel size. In all imaging experiments, the laser power and exposure time of the camera were set such that the full bit depth (12bit) of the camera was used.

### 3.4. Reconstruction algorithm

**Fig. 3:**
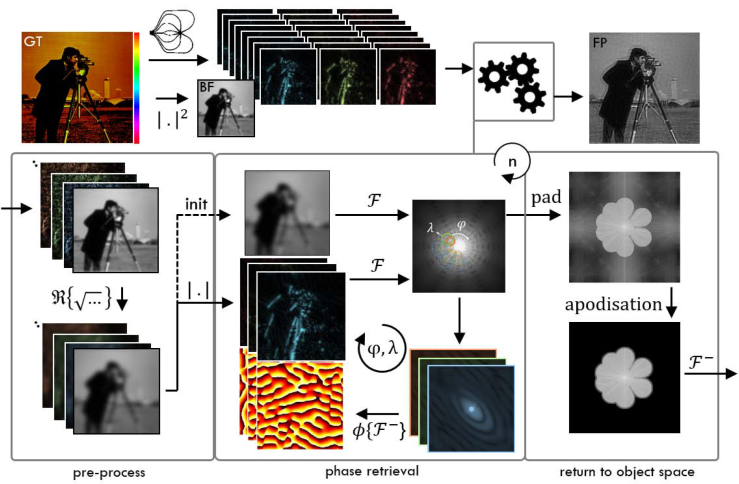
Phase-retrieval algorithm on simulated data. Details are provided in the text.

As our technique is based on Fourier ptychography it uses a phase retrieval algorithm [10], which was slightly modified and is depicted in the box of Fig. 3

. The algorithm aims to invert the imaging pipeline and thus requires a detailed description of the image formation. Let the raw images be denoted as *i*_*k*_0_,*k*_*c*__ (*x*), with *k*_0_ being the illumination wave vectors’ lateral component, and *k_c_* the coherent cut-off frequency of the used wavelength, i.e. $k_{c}=\frac{{\mathrm{NA}}_{o}}{\lambda }$. Using a coherent imaging model on the sample’s complex field *s*(*x*), which is illuminated with plane waves featuring wave vector *k*_0_ and imaged by an objective characterized by the coherent point spread function *h_c_*(*x*), the coherent image formation equation reads

(2) $$i_{k_{0},k_{c}}(x)={\left|\left[s(x)\times \text{exp}(-ik_{0}\times x)\right]⊗h_{c}(x)\right|}^{2}.$$

In this equation, i is the imaginary unit, and ⊗ is the convolution operator. The coherent PSF *h_c_*(*x*) can be defined easily via its Fourier transform *H_c_*(*k*), which is described by a circle centred on the spatial frequency coordinate origin and with value one inside and zero outside a radius *k_c_*, the coherent cut-off frequency. The goal of the phase retrieval algorithm is to find the amplitude *a*(*x*) and phase *ϕ*(*x*) component of the complex sample *s*(*x*) = *a*(*x*) × exp(i*ϕ*(*x*)). Three pre-processing steps are performed: (1) the raw data is background corrected, (2) then low-pass filtered, and (3) finally an initial guess of the amplitudes *a*_*k*_0_,*k*_*c*__ (*x*) is made. In analogy to the approach of Zheng [10] this is done by subtraction of a background estimate value *b*, and multiplication of a low-pass filter defined by the support of the incoherent optical transfer function to the image spectra *I*_*k*_0_,*k*_*c*__ (*k*) to remove noise from outside the pass-band of the objective, i.e. beyond the incoherent cut-off spatial frequency. As the incoherent cut-off frequency is twice the coherent cut-off frequency, a scaled version of the coherent transfer function can be used, i.e. $H_{c}\left(\frac{k}{2}\right)$. Note that Fourier analogues of real space functions, obtained via Fourier transform *𝔉* will be denoted via capitalization, so e.g. *H_c_*(*k*) = *𝔉*{*h_c_*(*x*)}, with *k* being the spatial frequency coordinate. The inverse Fourier transform is written as *𝔉*^−^.

After low-pass filtering, the real part *ℜ* of the square root is taken to approximate the field distribution that formed the recorded intensities:

(3) $$a_{k_{0},k_{c}}(x)=ℜ\left\{\sqrt{{𝔉}^{-}\left\{𝔉\left\{i_{k_{0},k_{c}}(x)-b\right\}\times H_{c}\left(\frac{k}{2}\right)\right\}}\right\}.$$

The phase retrieval part of the algorithm is then initialized using the estimated amplitude of a brightfield image as starting guess *f*^0^(*x*) [29] for the high-resolution Fourier ptychography image. We note that in conventional FP any starting guess can be used [10]. In each iteration up to a total of *n* rounds, *f^j^*(*x*) is sequentially updated for all available coherent illumination wave vectors *k*_0_. The sequence is chosen such that the respective sub-sampled parts of Fourier space (which are centred around *k*_0_ and with radius *k_c_*), are *spiralling out* from lower to higher spatial frequencies. Formally in the algorithm, the individual updates are performed in three steps. First, a temporary low-resolution image *t^j^*(*x*) is calculated from the Fourier ptychography estimate *f^j^*(*x*) for the current respective illumination featuring wave vector *k*_0_, cut-off frequency *k_c_* and amplitude transfer function *H_c_* as

(4) $$t_{k_{0},k_{c}}^{j}(x)={𝔉}^{-}\left\{F^{j}(k-k_{0})\times H_{c}\right\}.$$

The phase Φ(*t*(*x*)) of the temporary low-resolution image *t^j^*(*x*) is taken as an estimate of the phase distribution *ϕ*(*x*) of the sample *s*(*x*). Hence, only the amplitude of *t^j^*(*x*) is updated, i.e. replaced by the estimated amplitude *a*_*k*_0_,*k*_*c*__ (*x*) of the respective pre-processed raw image

(5) $$t_{k_{0},k_{c}}^{j+1}(x)=a_{k_{0},k_{c}}(x)\times \text{exp}\left(\text{i}\Phi (t^{j}(x))\right).$$

The updated temporary image’s spectrum $T_{k_{0},k_{c}}^{j+1}(k)$ is successively used to replace the respective region in Fourier space of the Fourier ptychography image’s spectrum *F*^*j*+1^(*k*). This region is centred on *k*_0_ within a support area defined by the coherent transfer function *H_c_*(*k*) of that respective wavelength:

(6) $$F^{j+1}(k)=F^{j}(k)\times \left(1-H_{c}(k-k_{0})\right))+H_{c}\left(k-k_{0}\right))\times T_{k_{0},k_{c}}^{j+1}(k-k_{0}).$$

After each loop the lower spatial frequencies can be updated using an incoherent brightfield image in analogy to the updating step with evanescent illumination. Note that this step is different to conventional FP [10] and was implemented to gain some form of access to oblique illumination information, which is necessary to avoid reconstruction artefacts [30, 31]. After *n* full loops, the final Fourier ptychography image *f*(*x*) is produced via apodization and a successive inverse Fourier transform with enlarged Fourier support (potentially made to fit via additional zero-padding) to yield a smoother transform result

(7) $$f(x)={𝔉}^{-}\left\{\mathrm{apo}\left(\mathrm{pad}(F(k))\right)\right\}.$$

For comparison to brightfield data, an intensity image can be created via squaring of the amplitude part of the Fourier ptychography reconstruction. The presented algorithm was tested on simulated data, as displayed in the top of Fig. 3 for a ground truth (GT) input and a successful FP intensity reconstruction.

## 4. Experimental results

**Fig. 4:**
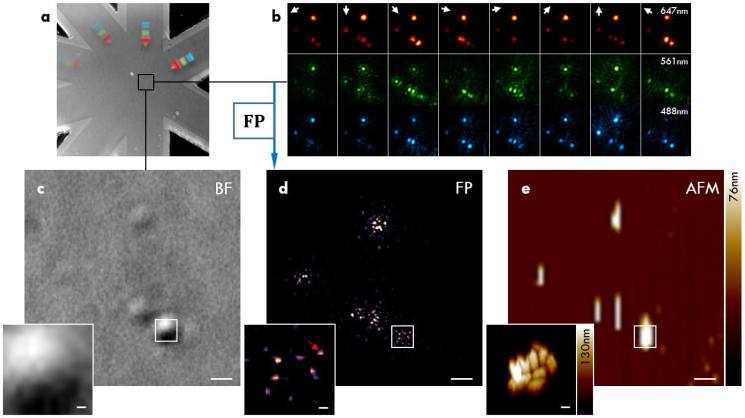
Imaging of metal-organic frameworks (MOFs) with FP. (a) Overview of the imaged region. (b) Raw evanescent scattering images under waveguide illumination with 488nm, 561nm, and 647nm laser light. (c) Brightfield image using sum of multiple LED wavelengths. (d) Intensity image created by Fourier ptychography. The red arrow in the inlay might be mistaken for individual particles but is most likely an image reconstruction artefact as its full width at half maximum (FWHM) is smaller then the theoretically achievable FWHM (∼ 50 nm versus ∼ 165 nm). (e) Atomic force microscopy image (line levelling artefacts prohibit a clear view of individual particles). Inlays show a zoomed region of a cluster of MOFs. The overview image (a) measures 100×100 μm^2^ and the scalebars in (c–e) are 1 μm and 100 nm in the inlays respectively.

**Fig. 5:**
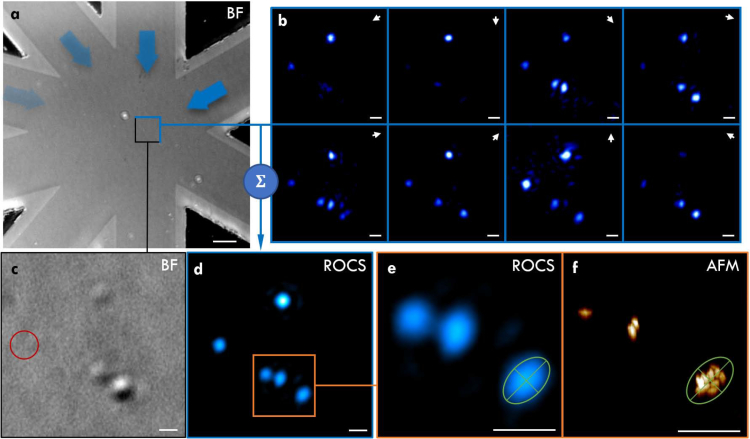
Imaging of metal-organic frameworks (MOFs) with ROCS. (a) Overview of the imaged region. (b) Raw evanescent scattering images under waveguide illumination with a 488 nm laser. (c) Brightfield image using sum of multiple LED wavelengths. The red circle highlights a cluster that is only visible under darkfield illumination. (d) Intensity image created by ROCS with (e) zoom onto MOF clusters. Although it is not possible to discern individual particles, the elongated shape of the clusters is visualised by ROCS in good agreement with (f) the atomic force microscopy image of the same region. The overview image (a) measures 100 × 100 μm^2^ and the scalebars in (b–f) are 1 μm.

To test waveguide-based FP experimentally, we imaged clusters of metal-organic frameworks (MOFs). The imaged MOFs belong to the group of Zirconium-MOFs with gold nano-rod core and have a small size distribution centered around 200 nm [32]. To ensure adherence of the MOFs to the waveguides, the waveguide chip was plasma-treated for 40 s at 40 W using a 0.35 mbar oxygen atmosphere. Then, a highly diluted aqueous solution of MOFs was drop-casted onto the waveguide chip imaging area and left to dry under a slight angle to provide a more even distribution of the particles. Note that the sample can be removed and the super-condenser cleaned for reuse via suitable sample-dependent solvents (e.g. acetone). Repeated plasma-treatment (required to aid sample adherence) will, however, destroy the single-mode characteristics of the waveguides. After imaging with the super-condenser, a ground truth image of the sample was generated via atomic force microscopy (AFM) using a commercial system (Bioscope RESOLVE, Bruker). The AFM was operated in tapping mode and RTESPA probes (Bruker) with a nominal spring constant of 6 N/m and resonant frequency 150 kHz. A line scanning resolution of 256 lines with 256 samples/line for 50×50 μm^2^ was used to generate an overview and 1168 lines with 1168 samples/line for 20×20 μm^2^ were used for greater detail of selected areas. To counter drift between frames, the individual raw frames were further aligned to each other using semi-automated alignment via the image processing software *line ROI image alignment* in Fiji [33]. As shown in Fig. 4

, we retrieved images displaying both enhanced contrast and features beyond the incoherent Abbe diffraction limit. In panel (a), an overview of the waveguide chip geometry is shown using a brightfield reflectance image. As shown in (c), the sample is only barely visible in brightfield incoherent illumination image (for the same objective), which displays an unresolved cluster of 370 nm size in terms of full width at half maximum of a Gaussian fit (data not shown). Displayed in panel (d), the underlying distribution of individual MOFs in this cluster is shown using atomic force microscopy (AFM) and finest scanning reveals dense clustering of particles. The same distribution of clusters that is visible in the AFM image is also present in the Fourier ptychography image in panel (c), which is, however, plagued by image artefacts that do not resolve individual gold nanorod cores in the clusters. An alternative method for reconstruction of evanescent darkfield scattering data is *rotating coherent scattering* (ROCS) microscopy [34], which is shown in Fig. 5

. Here, an image is simply generated by summation of the raw images over all azimuthal angles. Note that in ROCS no wavelength ’sweep’ as in waveguide-based FP is necessary but the achievable resolution gains are small (according to the Rayleigh criterion) [14]. It is found that the ROCS imaging procedure results in dramatic enhancement of image contrast (compare Fig. 5c and d), and individual clusters of MOFs can easily be discerned. Concurrently, it offers resolution in accordance with the Abbe criterion.

## 5. Discussion and conclusion

Care must be taken in interpreting the results of FP as the discernible spots produced by the phase retrieval algorithm cannot be associated to individual particles in the AFM recordings. For instance, although the clusters visible in FP can be mapped onto the AFM ground truth, the individual particles are slightly misplaced or even missing completely. Furthermore, the full width at half maximum (FWHM) of reconstructed ’individual’ particles is smaller then the theoretically achievable FWHM (∼ 50 nm versus ∼ 165 nm) and thus likely represents a reconstruction artefact. This can have multiple reasons. Firstly, the displacement might be traced back to drift of the sample during raw data acquisition. A shift of sample information even in the nanometer range might thus produce artefacts causing erroneous particle localization. Although drift was compensated for computationally, it cannot be avoided completely. Secondly, the gold core of the MOFs is asymmetric and causes scattering preferentially in certain directions. Paired with variable signal strength for different illuminations caused by higher losses of the waveguides in longer arms, this could lead to some particles *outshining* others in the reconstruction. Also note that the amplitude of the scattered light is wavelength dependent. Despite trying to adjust for this via variation of the laser illumination intensity and the exposure time of the camera, a limited maximum number of photons in certain raw frames restricted the achievable signal-to-noise ratio (SNR). Residual wavelength-dependent waveguide autofluorescence increases the challenge of limited SNR additionally. In the future, more advanced algorithms originally developed for conventional Fourier ptychography could be adopted to computationally alleviate some of these concerns [35]. Furthermore, axial chromatic offset could not be accounted for, which might stem from residual imperfections of the employed apochromatic objective and achromatic tube lens. This might be tackled via fine z-stepping of the objective (not possible in the presented set-up which offers no finer than 0.5 μm steps) and post-acquisition alignment or pre-acquisition calibration (potentially using multiple cameras to increase acquisition speeds). An additional complication is posed by the necessary image reconstruction, which had to be based on evanescent illuminations as well as a single bightfield image. In simulations, this restriction in terms of raw data was found to be *only* sufficient for artefact-free *intensity* reconstructions but had limited potential to extract the sample’s phase [31]. The algorithm is further especially challenged when noise-corrupted data is used or when only little overlap of spectral information between raw images is present. Still, the produced data is promising for the young field of labelfree chip-nanoscopy. In contrast, the processing procedure of ROCS allowed visibly artefact-free imaging, albeit with less resolution.

**Fig. 6:**
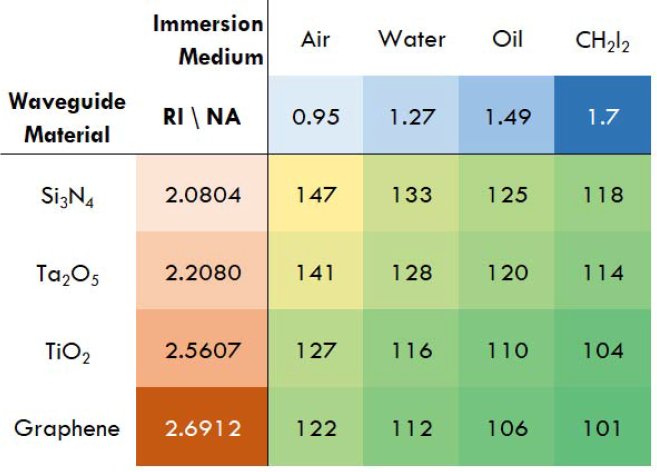
Theoretically achievable resolution given in nm via different waveguide materials and substrate/immersion objective combinations (assuming shortest illumination wavelength of 445 nm).

Looking ahead, a multitude of further developments are thinkable, both conceptually and practically. An example would be a change of the chip material. The catch here is the unavailability of established production methods for single-mode waveguides of more exotic character. Nevertheless, suitable candidates for alternative waveguide materials that also keep propagation losses low at short wavelengths are Ta_2_O_5_ [36], TiO_2_ [37], or graphene [38]) - see Fig. 6

. TiO_2_ is an especially interesting material, as it transmits even in parts of the near ultra-violet range, which would allow sub-100nm resolution:

(8) $$\Delta x_{\mathrm{TiO}}=\frac{405\;\text{nm}}{\left(1.49+2.66\right)}=97.6\;\text{nm}.$$

A further current conceptual bottleneck, the limited field of view, could be alleviated through use of a *slab* region at the imaging area illuminated by un-tapered single-mode waveguides. Although initially propagating as *circular* waves (the two-dimensional analog of spherical waves produced by free-space point sources), the waves emanating from the waveguide outlets would be sufficiently close to plane waves already after a travelled distance of few wavelengths and thus suitable for quasi-coherent imaging. For a further field of view enhancement, illumination from a limited number of directions (potentially even only single-sided) could be feasible for amplitude-only samples [39] and would simplify both waveguide geometry and deliverable powers to the imaging area due to reduced bending losses. More speculatively, on-chip lasers [40] are an option to simplify the microscopy set-up and circumvent coupling losses. An intriguing alternative could further be the use of a broadly emitting fluorescent film to generate the illumination light with successive narrow-band emission filtering as proposed by Pang et al [30]. Although the illumination in this set-up is not coherent, FP algorithms have been developed that can be applied [29]. To tackle drift of the sample during image acquisition, a fully automated coupling procedure might speed up slow manual coupling of various wavelengths and to different inputs. A possible future improvement in this respect is input-multiplexing which is feasible via conventional fiber-array adaptors. These are standard in the telecommunication industry but are to date mostly available for infrared wavelengths.

In conclusion, we have developed a new microscopy illumination scheme for labelfree nanoscopy that combines coherent imaging with waveguide microscopy to realize a super-condenser. The waveguide geometry allows the use of maximally inclined coherent darkfield illumination and additionally makes use of the large refractive index of Si_3_N_4_ as waveguide material to further double the illumination wave vector amplitudes as compared to air. We validated our method in silico and tested it in experimental imaging of metal organic frameworks that contained gold nano-rod cores. As shown by atomic force microscopy, we were able to image MOF clusters successfully with ROCS and found that FP produces images with visible image artefacts. Taken together, Fourier ptychography in combination with enlarged illumination wave vectors can be a promising avenue to enable nanoscopic imaging without the requirement of extrinsic labels as long as the reconstruction procedures can be improved. The more robust ROCS processing presents meanwhile a suitable alternative and could potentially be used in conjunction with fluorescence on-chip superresolution microscopy [41, 42]. As such a combination would consist purely of widefield imaging techniques, a considerable increase in throughput is achievable as compared to scanning approaches like nearfield scanning optical microscopy.
